# Associations between cognitive enhancement therapy in old age and temporal‐spatial orientation and language: A case study

**DOI:** 10.1002/alz.090860

**Published:** 2025-01-09

**Authors:** Antonia Tziannarou, Constantinos Christodoulides, Andrea Hadjiloizou, Sotiria Moza

**Affiliations:** ^1^ Materia Group, Nicosia Cyprus; ^2^ Noesis Cognitive Center & Tech Solutions Ltd, Nicosia Cyprus

## Abstract

**Background:**

An 84‐year‐old male, with 16 years of education (retired physics teacher) was admitted to a long‐term rehabilitation centre to receive daily care, with cognitive decline symptoms. This study presents the data from a three‐month cognitive enhancement program.

**Methods:**

In September 2023, the patient underwent the Mini Mental State Examination (MMSE) in Greek, followed by cognitive enhancement sessions (45 minutes, twice a week) for three months. The sessions included exercises such as, reading the calendar, telling the time, planning daily activities, virtual travel videos and media, constructive tasks with cubes, language tasks (e.g., arranging words in the correct order to form sentences, finding synonyms and antonyms, and other). An alternative version of the MMSE was administered in January 2024. The patient’s medication regimen for dementia, depression, cholesterol, and hypertension, had remained unchanged over the last 6 months.

**Results:**

In the preliminary assessment, the patient scored 15/30 on the MMSE. He scored 0/10 on the temporal/spatial orientation task, 0/3 on the delayed recall task, 2/3 on the 3‐command language item, and 0/1 on the language repetition item. Following the cognitive enhancement program, he achieved 20/30 points on MMSE and specifically 3/10 on orientation, 3/3 on the 3‐command item, and 1/1 on the repetition item.

**Conclusions:**

The findings suggest that participating in targeted cognitive stimulation exercises may be linked to positive effects on orientation and language among older individuals, particularly, those with dementia. Our results could be influenced by the novelty of the stimuli in the patient’s life, potentially elevating cognitive demand temporarily and reaching a plateau over time. The study is ongoing to further explore these aspects.

